# Androgen receptor is negatively correlated with the methylation-mediated transcriptional repression of miR-375 in human prostate cancer cells

**DOI:** 10.3892/or.2013.2810

**Published:** 2013-10-24

**Authors:** MINGLIANG CHU, YUNLI CHANG, PING LI, YANJING GUO, KAIQING ZHANG, WEIQIANG GAO

**Affiliations:** 1 State Key Laboratory of Oncogenes and Related Genes, Stem Cell Research Center, Ren Ji Hospital, School of Medicine, Shanghai Jiao Tong University, Shanghai 200127, P.R. China; 2 Med-X Research Institute, Shanghai Jiao Tong University, Shanghai 200030, P.R. China

**Keywords:** androgen receptor, DNA methylation, miR-375, prostate cancer

## Abstract

Androgen receptor (AR) plays a critical role during the development and progression of prostate cancer in which microRNA miR-375 is overexpressed and correlated with tumor progression. Although DNA methylation is a key mechanism for the repression of gene expression, the relationship between AR and the expression or the hypermethylation of miR-375 is unknown. In this study, we found that AR-positive prostate cancer (PCa) cells showed high expression levels and hypomethylation of the miR-375. In contrast, AR-negative PCa cells displayed low levels and hypermethylation of the miR-375. Addition of 5-Aza-2′-deoxycytidine, a specific inhibitor of DNA methylation, into the culture medium reversed the low expression levels of miR-375 in the AR negative PCa cells. In addition, the total activity levels of DNA methyltransferases (DNMTs) were high in AR-negative PCa cells, in which hypermethylation of miR-375 promoter and low expression levels of miR-375 were observed. Taken together, these findings indicate that the negative correlation between AR and total DNMT activity is one of mechanisms to influence the methylation status of miR-375 promoter, which in turn regulates the expression of miR-375.

## Introduction

Prostate cancer (PCa) is the most frequently diagnosed male cancer in the developed world with increasing rates in the developing countries (1). DNA methylation of the promoter region is one of the regulatory mechanisms of gene expression in prostate cancer (2). Not only tumor-suppressor genes, but also numerous miRNAs can be silenced by methylation of the relavant promoters in prostate cancer (3).

microRNAs (miR) are a conserved class of small non-coding RNAs (approximately 22 nucleotides) which usually cause gene silencing via translational repression or degradation of specific mRNA (4). microRNA genes are frequently located in cancer-associated genomic regions or at certain fragile sites. Their aberrant expression has been shown to be significantly involved in human cancer (5–7). In particular, miR-375 was initially believed to be a tumor suppressor, as it targets certain oncogenes and its expression levels are significantly low in most tumors, including esophageal squamous cell carcinoma (8), oral squamous cell carcinoma (9), gastric carcinomas (10), pancreatic cancer (11), hepatocellular carcinoma (12), melanoma (13), squamous cervical cancer (14) and head and neck squamous cell carcinomas (15).

However, recently studies have indicated that miR-375 is overexpressed in prostate and breast cancers, suggesting that it might exert an oncogene function in these two cancer types (16,17). Both breast cancer and prostate cancer are sex hormone-dependent for their growth and progression, and are also remarkably similar in some physiological and pathological phenomena (18). For example, previous studies have shown that estrogen receptor α (ERα), a female hormone recentor, is involved in the DNA hypomethylation and the expression of miR-375 in breast cancer cells (17). Androgen receptor (AR) is a male hormone receptor, which plays a crucial role in the initiation and growth of prostate cancer (19). However, the relationship between androgen receptor and DNA methylation and the expression of miR-375 in prostate cancer cells is not yet known and therefore the subject of the present study. We report that AR is negatively correlated with the methylation-mediated transcriptional repression of miR-375 in human prostate cancer cells.

## Materials and methods

### Cell lines

LNCaP, 22Rv1, PC-3 and DU145 cell lines were obtained from the American Type Culture Collection (ATCC, Manassas, VA, USA). C4-2 cells were obtained from UroCor (Oklahoma City, OK, USA). PC3/AR cells are a stable cell line which were transfected with a plasmid containing the AR cDNA sequence; PC3/neo cells contained the identical vector lacking the AR cDNA sequence (20). Cells were maintained according to the protocols of the manufacturer and provider. In the NCBI/GEO website, the GDS1699 database is from AR-positive PCa cells (LAPC-4, MDA2a, MDA2b, LNCaP, 22RV1 cells) and AR-negative PCa cells (PPC-1, PC3 and DU145 cells).

### Quantitative real-time PCR analysis of miRNA expression

Total RNA were extracted using the RNeasy Plus Mini kit (Qiagen, Valencia, CA, USA) from various PCa cell lines. Reverse transcription was carried out using the TaqMan MicroRNA Reverse Transcription kit (Applied Biosystems, Foster City, CA, USA). Relative quantities of miR-375 were performed with a 7500 Real-Time PCR System (Applied Biosystems) by using TaqMan^®^ microRNA Assays (Applied Biosystems). Gene expression was normalized by the endogenous control RNU44, and the Ct values were calculated using the ΔΔCt method.

### RNAi

Specific AR shRNA sequence (5-GGTGTCACTATG GAGCTCTCA-3) was designed as previously described (21). AR shRNA was cloned into the pLKO.1- scramble cloning vector (Addgene plasmid 10879). Procedures of vector construct and lentiviral production and infection were according to the online protocol: http://www.addgene.org/tools/protocols/plko/#B. At 72-h post-infection, total RNA and bisulfite conversion DNA were prepared from LNCaP AR RNAi cells (LNCaP-sh-AR) and RNAi control cells (LNCaP-sh-control).

### Cytospin and immunofluorescence

For immunocytochemical analysis of LNCaP AR RNAi cells (LNCaP-sh-AR) and RNAi control cells (LNCaP-sh-control), 1×10^4^ infected cells (72 h) were washed and resuspended in 200 μl of phosphate-buffered saline (PBS). The preparation cells were spun down onto coated slides using a ThermoShandon Cytospin 4 apparatus (Thermo Shandon Inc., Pittsburgh, PA, USA) at 1500 rpm for 5 min. The slides were then air-dried for 5 min and fixed for 10 min in 4% paraformaldehyde (Dingguo Biotechnology Co., Ltd., Beijing, China) at room temperature. The fixed cells were washed three times with PBS and incubated for 10 min in 0.5% Triton X-100. Cells were washed carefully and incubated for 30 min in 10% goat serum and then for overnight at 4°C with a primary rabbit anti-androgen receptor antibody (Epitomics, Burlingame, CA, USA) and subsequently with secondary Alexa Fluor 488-conjugated donkey anti-rabbit IgG (Molecular Probes, Eugene, OR, USA). Coverslips were mounted with VectaShield mounting media containing 4,6-diamidino-2-phenyindole, dilactate (DAPI) (Vector, Burlingame, CA, USA). Epifluorescence microscopy was performed with a Nikon microscope (Nikon, Melville, NY, USA).

### Sodium bisulfite modification and sequencing

Cells were directly subjected to bisulfite conversion by using the EZ DNA methylation-direct kit (Zymo Research, Irvine, CA, USA) as per manufacturer's protocol. The forward primer: 5-GGGGATTGAATAGGTAGTATAAG-3 and reverse primer: 5-ATAATCTCCTAATCCTAATCTTCC-3 were used for miR-375 bisulfite PCR. Ex Taq HS DNA polymerase (Takara Biotechnology Co., Ltd., Dalian, China) were employed in PCR amplification, and the conditions were 95°C, 5 min, 39 cycles (95°C, 30 sec; 58°C, 30 sec; 72°C, 30 sec), 72°C, 7 min. The PCR products were then ligated into the pMD19-T Simple Vector (Takara Biotechnology Co.). Colonies (n=10) were selected per PCa cell line and sequenced by Shanghai Shenggong Biotech (Shanghai, China).

### Treatment of cells with 5-Aza-2′-deoxycytidine

The DU-145 and PC-3 cells were plated in T-25 flask. Subconfluent cells (60–70% confluent) were maintained in medium supplemented with 5 μM of 5-Aza-2′-deoxycytidine (5-Aza-dC) (Sigma-Aldrich, St. Louis, MO, USA) and cells treated with vehicle (dimethylsulfoxide, DMSO) served as control. The cells were incubated for 5 days with a change of culture medium every 24 h.

### DNA methyltransferase analysis of PCa cells

PCa cell nuclear extracts were prepared using the nuclear and cytoplasmic protein extraction kit (Bioteke Corp., China) in accordance with the manufacturer's protocol. Total DNA methyltransferase activity was assayed using an EpiQuik DNA Methyltransferase Activity/Inhibition Assay kit (Epigentek, Brooklyn, NY, USA), according to the manufacturer's protocol. The relative expression values of DNMT1 (GDS1699/38832), DNMT3A (GDS1699/21176), DNMT3B (GDS1699/16487) were obtained from the NCBI GEO website.

### Data analysis

Data were analyzed using the SPSS 13.0 software (SPSS Inc., Chicago, IL, USA) and Prism GraphPad 5 (GraphPad Software, La Jolla, CA, USA). Statistical analysis was performed using, paired and unpaired sample t-test. p-values <0.05 were considered significant.

## Results

### Expression levels of miR-375 are higher in PCa AR-positive cell lines than in PCa AR-negative cell lines

**Figure 1:**
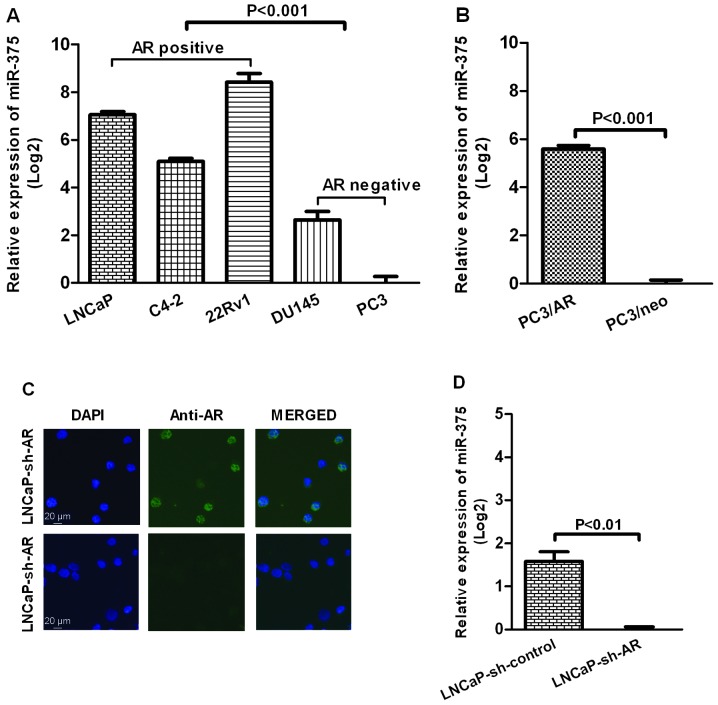
The expression levels of miR-375 in PCa cell lines. (A) LNCaP, C4-2 and 22RV1 cells are AR-positive PCa cell lines; DU145 and PC3 cells are AR-negative PCa cell lines. (B) PC3/AR cells are a stable cell line stably transfected with a plasmid containing the coding region of the human AR; PC3/neo cells contain the identical vector lacking the AR cDNA sequence. (C) LNCaP cells were treated with lentivious AR shRNA (LNCaP-sh-AR) and control shRNA (LNCaP-sh-control). Seventy-two hours after infection, cells were stained with an anti-AR antibody (green) and DAPI (blue). (D) LNCaP cells were treated with lentivious AR shRNA (LNCaP-sh-AR cells) or a control shRNA (LNCaP-sh-control). Data are presented as log2 value of miR-375 in PCa cell lines normalized to (A) PC3 cells, (B) PC3/neo cells, and (D) LNCaP-sh-AR cells. Relative expression of miR-375 in PCa cell lines was determined by qRT-PCR and corrected to RUN44 levels. Results are representatives of three independent experiments. Data are shown as mean ± SEM. Error bars represent standard deviations.

To examine the expression levels of miR-375 in PCa cell lines, several AR-positive cell lines (LNCaP, C4-2, 22Rv1) and AR-negative cell lines (PC3 and DU 145) were analyzed by real-time PCR. The results revealed that the expression levels of miR-375 were significantly higher in PCa AR-positive cell lines than in PCa AR-negative cell lines (p<0.001, Fig. 1A). Additionally, PC3/AR cells, which are PC3 cells engineered to express AR (20), showed a high expression level of miR-375 (p<0.001, Fig. 1B). While knocking down of AR by lentivious AR shRNA interference in LNCaP cells (Fig. 1C) reversed the high level of miR-375 (p<0.01, Fig. 1D).

### microRNA-375 promoter shows a hypermethylation phenotype in AR-negative PCa cells

To determine whether DNA methylation was involved in the regulation of miR-375 gene expression, the 1 kb upstream of miR-375 gene was scanned for potential CpG islands using the Li Lab program at http://www.urogene.org/cgi-bin/methprimer/methprimer.cgi (22).

**Figure 2:**
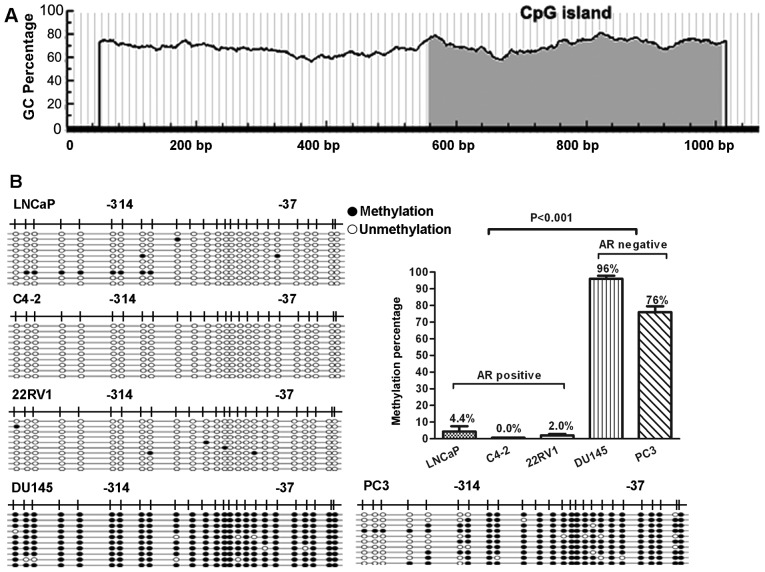

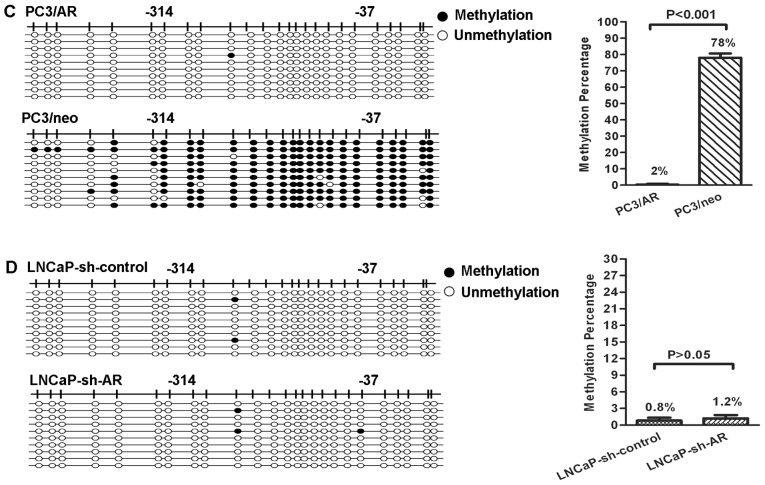
Methylation status of miR-375 CpG island. (A) A CpG island is predicted on the 1 kb upstream of miR-375 gene. CpG island is indicated as grey area. (B–D) Schematic summary of 25 CpG sites in the miR-375 promoter region from -314 bp to -37 bp. Methylation analysis was shown in 10 clones from each cell line. Each circle represents a single DNA methylated or demethylated site, and each row of circles represents a single clone. The methylation percentages of 10 clones from each of the cell lines are summarized in the bar chart on the right. Data are shown as mean ± SEM. Error bars represent standard deviations.

This analysis revealed a CpG island in the region of miR-375 promoter (Fig. 2A). To investigate the methylation status of the island, the genomic DNAs of PCa cells were isolated and pyrosequenced using bisulfite sequencing (BSP). We found that the CpG islands in the miR-375 promoter region were hypermethylated (76–96%) in AR-negative PCa cells (PC3 and DU145), but only very limited methylation (0.0–4.4%) was seen in AR-positive PCa cells (LNCaP, C4-2, 22Rv1) (p<0.001, Fig. 2B). In addition, PC3/AR cells also showed a very low methylation (0.4%) status in the miR-375 promoter region, while the PC3-neo cells remained hypermethylation (78%) status in the miR-375 promoter region (p<0.001, Fig. 2C). However, knocking down AR by lentivious shRNA interference in LNCaP cells did not change the hypomethylation status of miR-375 promoter (p>0.05, Fig. 2D).

### 5-Aza-dC reversed the expression of miR-375 in AR-negative DU145 and PC3 cell lines

**Figure 3:**
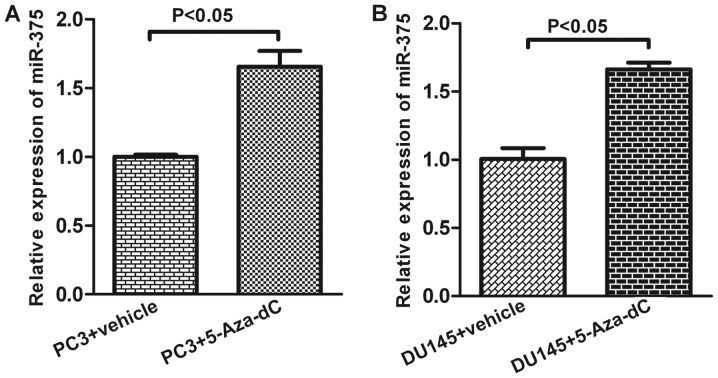
The relative expression of miR-375 in PC-3 and DU145 cell lines after treatment with 5-Aza-dC. PC-3 and DU145 cell lines were treated for 5 days with 5 μM 5-Aza-dC or vehicle. Results are representative of three independent experiments. Data are shown as the mean ± SEM. Error bars represent standard deviations.

To verify whether the low expression level of the miR-375 resulted in the promoter hypermethylation status of the AR-negative PCa cells, a demethylating agent 5-Aza-dC was used to treat AR-negative PC-3 and DU145 cells. Real-time PCR analysis revealed an elevation in the expression of miR-375 in these cells 5 days post 5-Aza-dC (5 μM) treatment (p<0.05, Fig. 3).

### Expression of DNA methyltransferase enzymes in PCa cell lines

**Figure 4:**
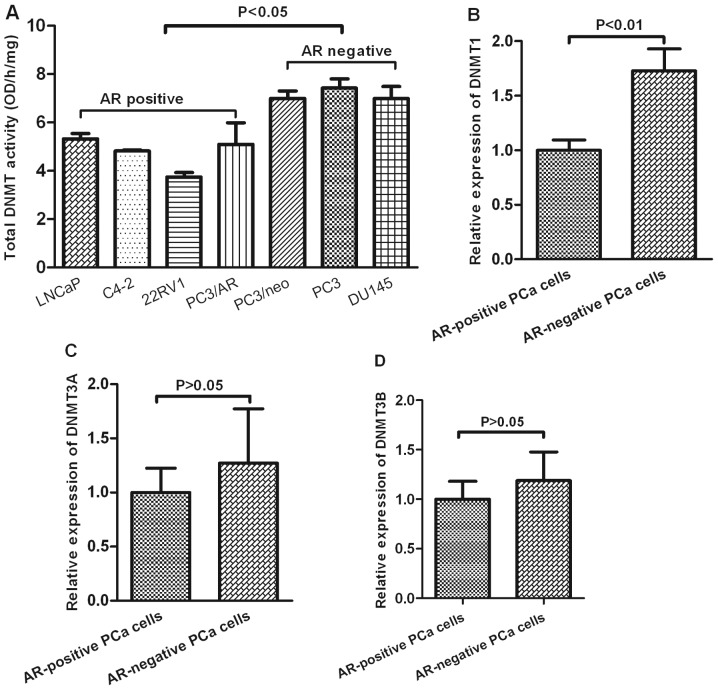
Analysis of activities of DNA methyltransferase enzymes in AR-positive PCa cell lines and negative PCa AR- cell lines. (A) Results from triplicates of total activity levels of DNMTs (as measured by an ELISA-based DNMT activity assay) are shown. (B–D) The data are based on the study GDS1699 from the NCBI/GEO database. AR-positive PCa cells include LAPC4, MDA2B, LNCaP, 22RV1 and MDA2A cells; and AR-negative PCa cells include PPC, PC3 and DU145 cells. Relative expression of (A) DNMT1, (B) DNMT3A, (C) DNMT3B in PCa cell lines were normalized to AR-positive PCa cells. Data are shown as mean ± SEM. Error bars represent standard deviations.

To study whether the DNA methyltransferase enzymes were involved in the promoter methylation status of miR-375, the total activity levels of DNMTs were measured by an ELISA-based DNMT activity assay. As shown in Fig. 4A, total DNMT activity was higher in AR-negative PCa cells (PC3/neo, PC3 and DU145) than in AR-positive PCa cells (LNCaP, C4-2, 22Rv, PC3/AR) (p<0.05, Fig. 4A). We further analyzed the expression of DNMT1, DNMT3A and DNMT3B in PCa cell lines based on the study GDS1699 from the NCBI/GEO database. This analysis revealed that the levels of DNMT1 were much higher in AR-negative PCa cells than in AR-positive PCa cells (p<0.01). However, there was no significant difference in the expression of DNMT3A and DNMT3B between AR-positive PCa and AR-negative PCa cells.

## Discussion

We have presented evidence that AR-negative PCa cells have high activity levels of total DNMT, hypermethylation of the miR-375 promoter and lower expression levels of miR-375. Yet, these results were exactly reversed in AR-positive PCa cells. Hence, we propose that the total DNMT activity is negatively regulated by the AR, which results in hypomethylation or hypermethylation of the miR-375 promoter in AR-positive PCa cells or AR-negative PCa cells, respectively. The methylation-mediated transcriptional repression then determines the different expression level of miR-375 in these PCa cells.

The growth and progression of prostate cancer depend largely on AR signaling (21). AR is a key transcription factor which is activated by androgens and transduces androgen signaling in prostate cells (21). In prostate cancer, many oncogenic genes such as prostate-specific antigen (PSA) (23), Cdk1 and Cdk2 (24), PMEPA1 (25), FGF8 (26) as well as TMPRSS2 (27) are closely associated with AR. A recent study has shown that miR-375 is an oncogenic miRNA and targets the Sec23A tumor suppressor gene in prostate cancer (16). However, it is unclear how AR interacts with miR-375. Our results demonstrate that the AR status can influence the expression of miR-375. While AR-negative PCa cells show a low level of miR-375, AR-positive PCa cells display a high level of miR-375. Overexpression of AR in PC3 cells upregulates the level of miR-375, while knockdown of AR in LNCaP cells downregulates the level of miR-375.

Given that the hypermethylation of DNA is one of the important mechanism to silence gene expression, we further investigated whether the low expression level of miR-375 in AR-negative PCa cells was due to the effect of methylation. Our findings indicate that the promoter of miR-375 is hypermethylated in AR-negative PCa cell lines. In contrast, the promoter of miR-375 is hardly methylated in AR-positive PCa cells. Overexpression of AR in PC3 cells reversed the hypermethylation of the miR-375 promoter. This differential methylation patterns in AR-negative and AR-positive cells were correlated with the expression of miR-375. These results suggest that DNA methylation maybe one of the important factors to silence the miR-375 gene expression in AR-negative PCa cells. The conclusion is further confirmed by our finding that the low expression level of miR-375 could be reversed through using a demethylating agent 5-Aza-dC in AR-negative DU145 and PC3 cells that otherwise show a hypermethylation pattern.

It should be pointed out that unexpectedly, knockdown of AR in LNCaP cells did not change the methylation status of miR-375. The cells still retain a hypomethylation status after AR knockdown. This might have been due to the lentivious AR shRNA that could be only transient in LNCaP cells, which are highly dependent on AR; yet, the PC3/AR is a stable cell line that has been developed for a long time (20). Additional studies are needed to clarify this point.

DNA methylation is mediated by a family of DNA methyltransferase enzymes (DNMTs). It is well known that DNMT overexpression induces aberrant hypermethylation, which contributes to the silence of genes in various cancer cells (28–31). Our studies also show that AR-negative PCa cells, with a hypermethylator status of the miR-375, have a high level of total DNMT activity. Among these multiple DNMT isoforms, DNMT3A and DNMT3B are responsible for creating global DNA methylation patterns during embryogenesis and gametogenesis (32), yet Dnmt1 is in charge of maintaining the established DNA methylation patterns (33). From the NCBI/GEO database, we further found that the DNMT1, but not DNMT3A and DNMT3B, was overexpressed in AR-negative PCa cells. According to the above results, we propose that elevated total DNMT, especially DNMT1, may play an important role in the hypermethylator phenotype in human AR-negative PCa cell lines. Nevertheless, the PCa cell lines used in NCBI/GEO database are not exactly the same as in our experiments. Hence, this hypothesis needs further confirmation.

In conclusion, this study indicates that the AR regulated expression level of DNMTs is likely one of the mechanisms to influence the methylation status of the miR-375 promoter, which in turn regulates the expression of miR-375.
